# Altered Microbial Composition of Drug-Sensitive and Drug-Resistant TB Patients Compared with Healthy Volunteers

**DOI:** 10.3390/microorganisms9081762

**Published:** 2021-08-18

**Authors:** Fernanda Valdez-Palomares, Marcela Muñoz Torrico, Berenice Palacios-González, Xavier Soberón, Eugenia Silva-Herzog

**Affiliations:** 1 Laboratorio de Vinculación Científica, Facultad de Medicina-UNAM en INMEGEN, Mexico City 14610, Mexico; palomares.fernanda@gmail.com (F.V.-P.); bpalacios@inmegen.gob.mx (B.P.-G.); 2 Instituto Nacional de Enfermedades Respiratorias, Mexico City 14080, Mexico; dra_munoz@hotmail.com; 3 Departamento de Ingeniería Celular y Biocatálisis, Universidad Nacional Autónoma de México (UNAM), Cuernavaca 62210, Mexico; soberon@ibt.unam.mx

**Keywords:** *Mycobacterium tuberculosis*, lung microbiota, healthy individuals, core sputum microbiota

## Abstract

*Mycobacterium tuberculosis* infection has three discernible outcomes: active tuberculosis, latent tuberculosis, or clearance of the bacterium. The outcome of the infection depends on the interaction of the bacterium, the immune system, and the microbiome of the host. The current study uses 16S rRNA sequencing to determine the diversity and composition of the respiratory microbiome of drug-resistant and drug-sensitive tuberculosis patients as well as healthy volunteers. Tuberculosis patients exhibited increased microbial diversity and differentially abundant bacteria than healthy volunteers. Compositional differences were also observed when comparing drug-sensitive or -resistant tuberculosis patients. Finally, we defined and assessed the differences in the core sputum microbiota between tuberculosis patients and healthy volunteers. Our observations collectively suggest that in sputum, *Mycobacterium tuberculosis* infection is related to altered bacterial diversity and compositional differences of core members of the microbiome, with potential implications for the bacterial pulmonary ecosystem’s stability and function.

## 1. Introduction

It is now widely accepted that persistent infections reflect an equilibrium between the host and the pathogen that is established and maintained by a network of interactions. These interactions include the pathogen, the host immune system, and its microbiome. These considerations apply to tuberculosis (TB), an infectious disease caused by *Mycobacterium tuberculosis* (*Mtb*), which has plagued humankind for centuries and remains a major global health problem. TB causes high morbidity and mortality worldwide: 45 million disability-adjusted life years (DALYs) and more than 1.5 million deaths were reported in 2019 [1]. Approximately 10% of those exposed to the bacilli develop active tuberculosis, while the remaining 90% either clear the bacteria or harbor them in a non-replicating state called latent tuberculosis [2]. What determines the outcome is not entirely known, but interactions with the host immune system and microbiome are expected to be important factors [3,4].

The development of high-throughput sequencing technologies has revealed the importance of microbiota in several biological processes and the development and progression of infectious diseases, gastrointestinal cancers, and metabolic, respiratory, and autoimmune diseases [5,6,7,8,9,10,11,12,13,14]. The study of the microbiome in the respiratory tract has lagged with respect to other anatomical sites due, in part, to the difficulty of obtaining samples and the earlier notion that the lung was a sterile compartment. However, in the last decade, evidence of the role of the microbiome in respiratory tract illnesses, including cystic fibrosis, chronic obstructive pulmonary disease (COPD), asthma, pneumonia, and TB, has increased [15,16,17].

Studies on respiratory microbiota in TB are limited, and even fewer comparative studies on healthy and infected subjects have been reported [4,18,19,20,21,22]. The present study used sputum samples from patients and induced sputum from healthy volunteers to minimize the difference in the sampling of both groups. This study aims to characterize the microbiome of active drug-sensitive or -resistant TB patients and compare them to healthy individuals (HVs).

## 2. Materials and Methods

### 2.1. Study Population and Clinical Data

Tuberculosis patients and healthy volunteers were recruited from the National Institute of Respiratory Diseases (INER) and the National Institute of Genomic Medicine (INMEGEN) in Mexico City. Sputum samples were collected from TB patients, whereas induced sputum was obtained from healthy volunteers after nebulizing with hypertonic saline solution, which produces coughing. TB patients with COPD were excluded. Both sputum samples may contain a mixture of oral, pharyngeal, and lung microbiota. Microbiology sputum smears and culture were performed on patient samples at the microbiology laboratory of the INER. All participants signed a voluntary informed consent before we obtained a sputum sample. The project was approved by the Ethics Committee of INMEGEN (CEI2017/21) and followed the principles of the Declaration of Helsinki.

### 2.2. Sample Collection and DNA Extraction

Sputum samples from TB patients and induced sputum from HVs were heat-inactivated for 20 min at 80 °C, pretreated with 500 mg of *N*-acetyl-l-cysteine for 15 min and two volumes of NaOH 2.0% for 10 min, with two intermediate shaking sessions. PBS was added to the mixture for a final volume of 50 mL, and two steps of centrifugation were performed. First, the pellet was resuspended with 10 mL of deionized sterile water and incubated for 30 min. After the second centrifugation, the sample was resuspended in 1.5 mL deionized sterile water. DNA extraction was performed using the QIAamp UCP pathogen kit (Qiagen, Hilden, Germany,) following the manufacturer’s instructions. Purified DNA was diluted to a final concentration of 50 ng/µL and stored at −20 °C. DNA integrity was verified by agarose gel electrophoresis and quantified spectrophotometrically using Nanodrop (Thermo Scientific, Wilmington, DE, USA).

### 2.3. Amplification of 16S rRNA Hypervariable Regions and Library Preparation

The overall respiratory microbiota composition was analyzed using 16S rRNA gene sequencing. Hypervariable regions V3–V4 were amplified by PCR using V3f (TCGTCGGCAGCGTCAGATGTGTATAAGAGACAGCCTACGGGNGGCWGCAG) and V4r (GTCTCGTGGGCTCGGAGATGTGTATAAGAGACAGGACTACHVGGGTATCTAATCC) primers with Illumina adaptors, following the manufacturer’s recommendation (16S Metagenomic Sequencing Library Preparation, Illumina, San Diego, CA, USA). Amplification of the target sequences was performed in two steps, as recommended. After amplification, the samples were analyzed by TAPESTATION and quantified using QUBIT. Samples were pooled in equimolar amounts at a final concentration of 12 pM and sequenced using the 500-cycle MiSeq V2 kit (Illumina, San Diego, CA, USA); 10% pf PhiX was used to increase diversity and improve sequencing. No DNA samples were used as controls, and no amplification product was detected after the first or second step of amplification, indicating minimal or no bacterial contamination.

### 2.4. Sequencing Data Analysis and Diversity Comparisons

Illumina fastq reads were processed using the Quantitative Insights Into Microbial Ecology 2 (QIIME 2) software package [23]. Denoising quality, chimera check, and clustering were performed using the Divisive Amplicon Denoising Algorithm 2 (DADA2) plugin tool and denoise-paired instruction. The resultant Amplicon Sequence Variant (ASV) table is a higher-resolution analog of the traditional OTU table; ASVs can be resolved exactly down to the level of single-nucleotide differences over the sequenced gene region. For taxonomic annotation, the SILVA database (release_138) was used as the reference 16S database, together with the naïve-Bayes-algorithm-based trained classifier for a taxonomic assignment at 97%, using feature classifier classify-sklearn instructions. Initial pre-processing of the ASV feature table was conducted using the Phyloseq package [24]. We applied rarefaction at 90% of minimum sampling depth (Figure S1), and a rooted phylogenetic tree was generated for further diversity comparisons using Phyloseq and Vegan R packages. Alpha (within-sample) diversity was measured using Shannon’s, abundance-based coverage estimator (ACE diversity index), Chao1 diversity, Simpson’s, and Fisher’s indexes. The α diversity pairwise comparisons were made using the Wilcoxon rank-sum test with Holm’s *p*-value adjustment method. Beta (across-sample) diversity was measured using the Bray–Curtis dissimilarity statistic [25] that is based on compositional dissimilarity between samples, taking abundance, unweighted UniFrac distances that measure phylogenetic distances between taxa, and weighted UniFrac distances into account [26].

### 2.5. Relative Abundance, Core Microbiome, and Statistical Analysis

To identify bacterial taxa (ASVs) whose abundances significantly differ among TB and HV as well as DR and DS-TB patients, we applied the Analysis of Compositions of Microbiomes with Bias Correction (ANCOM-BC) methodology [27]. ANCOM-BC estimates the unknown sampling fractions and corrects the bias induced by the differences among samples. The sampling fraction is defined as the ratio of the expected absolute abundance of a taxon in a random sample to its absolute abundance in a unit volume of the ecosystem from where the sample was derived. The sample fraction is affected by the microbial load in a unit volume (mL of sputum) of the ecosystem and the library size of the corresponding sample. We introduced ‘Healthy’ or ‘TB diagnosis’ and ‘Age’ as sample-specific offset terms for sampling fraction estimation. In ANCOM-BC, the offset term serves as the bias correction, and the linear regression framework in log scale is analogous to log-ratio transformation to deal with the compositionality of microbiome data. To correct for multiple testing, we set the Benjamin–Hochberg (BH) FDR to 0.05. To evaluate the effect size associated with each taxon when comparing groups, 95% simultaneous confidence intervals for the mean DA of each taxon in the two experimental groups were adjusted for multiplicity using the FDR method. To assign an ASV as a member of the core microbiome, the most stringent definition requires its presence across all subjects sampled [28]; however, we cannot assume that the lack of an ASV corresponds to its true absence or that it is below the detection level in a small sample. Thus, we used a less stringent threshold to define a core ASV—being present in 95% at the phylum level and 80% at the genus level, with a relative abundance higher than 0.1%, across all samples. Statistical tests were performed in R version 3.6.7 and 4.0.0.

## 3. Results

### 3.1. Sequencing Data and Participant Demographics

We hypothesized that *M. tuberculosis* infection disturbs the pulmonary bacterial ecosystem, thus influencing the outcome of the disease. To analyze this, we determined the sputum microbiota of both HVs and TB patients. Sputum was collected from TB patients and induced sputum from 6 HVs. Sputum smears tests and cultures were performed on all patient samples. Out of the 41 patients, two were not confirmed with TB and were, thus, not included in the study. We obtained 19 positive smear subjects (3+): 3 with moderately positive smears (2+), 4 with weakly positive smears (1+), and 6 with negative smears and subsequent positive culture. The majority of TB patients (67%) and all HVs resided in the Mexico City metropolitan area; no significant correlation was found between place or residence and the microbiota composition of TB patients.

*M. tuberculosis* lineage data was available for 19 patients, of which 79% (*n* = 15) were L4-Harlem, 5% (*n* = 1) IndoOceanic_L1, 5% (*n* = 1) EAI_L3, and 10% (*n* = 2) *M bovis*. Therapeutic outcome data was available for only for 16 patients: 81% of them (*n* = 13) were cured and discharged, 6% were diseased (*n* = 1), and 12% abandoned treatment (*n* = 2). Clinical data of different therapeutic outcomes (i.e., death, recurrence, clearance), as well as co-morbidities (diabetes, hypertension, smoking habit) were insufficient to establish a correlation between microbiota composition and clinical outcome.

TB patients were classified drug-resistant (DR-TB) if they were mono-resistant, multidrug-resistant (MDR) and extensive drug-resistant (XDR) (*n* = 27 patients), and drug-sensitive (DS-TB) (*n* = 12 patients). Clinical and demographic characteristics are summarized in Table 1. Age and gender were not matched between TB patients and HVs; however, these factors were considered in the statistical analysis.

After quality control and chimera removal, 243,447 and 19,836 total sequences were obtained for TB patients and HVs, respectively, with an average of 5938 and 3306 readings per sample. Rarefaction allowed us to confirm that sufficient sequencing depth was obtained to describe the respiratory microbiome across all individuals (Figure S1). By grouping single readings from individual samples, 3888 independent ASVs were identified.

### 3.2. α and β-Diversity Differences Were Observed When Comparing Sputum Microbiota from Healthy Volunteers and TB Cases

**Figure 1:**
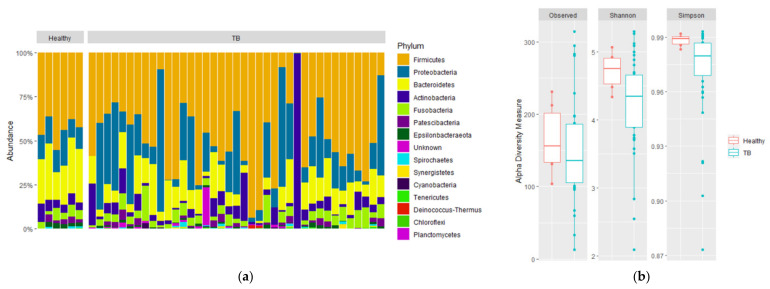
Composition and alpha diversity of sputum microbiota of healthy volunteers and TB patients. 16S rDNA from sputum samples were sequenced, and relative abundances for phyla are shown for (**a**) individual sample relative abundances of phyla by group: healthy volunteers, drug-sensitive TB, and drug-resistant TB. (**b**) Comparison of alpha diversity based on the number of observed species, Shannon diversity, and Simpson diversity of HVs (red) and TB patients (green). Statistical significance between groups is indicated by (*p* < 0.05; Wilcoxon test).

To gain further insight into the pathogenesis of the disease, we evaluated the microbial diversity of the respiratory microbiome since it is related to the immunity of the host and the virulence of the microbes [29,30]. First, the α-diversity at the phylum level of HVs and TB patients was analyzed (Figure 1a). Furthermore, HVs were dominated by fewer genera compared to TB patients, according to Simpson’s index (*p* = 0.022), although no statistical differences were observed for the other alpha diversity indexes (Figure 1b).

**Figure 2:**
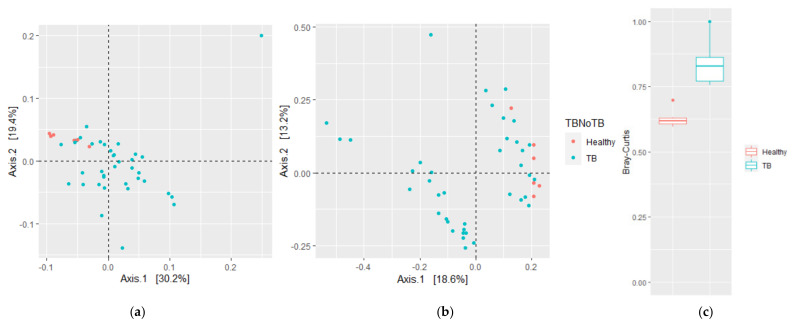
Community structure of sputum microbiota of HVs and TB patients. Principal coordinate analysis (PCoA) plots based on (**a**) weighted and (**b**) unweighted UniFrac distances show significant distinct bacterial community clusters between TB patient and HV lung microbiota. (**c**) Bray–Curtis dissimilarity index. Plots represent HV samples (red symbols) and TB patient samples (green). Statistical significance between groups in (**c**) is indicated by (*p* < 0.05; ADONIS test).

β-diversity was then evaluated using weighted and unweighted UniFrac analyses, which measure phylogenetic distances, considering or not the relative abundance in the sample, as well as the Bray–Curtis (BC) dissimilarity index, which measures compositional dissimilarity based on counts observed across groups. The HV samples were clustered together, suggesting a phylogenetically related bacterial community, while TB patients were more dispersed and clustered in two groups, one of them close to HVs (weighted UniFrac *p* = 0.004 (Figure 2a); unweighted UniFrac *p* = 0.003 (Figure 2b); BC *p* = 0.001 (Figure 2c)). Taken together, these results suggest that the altered diversity of the respiratory tract microbiota is related to *M. tuberculosis* infection and is defined by increased richness, abundance, and phylogenetic diversity.

### 3.3. Antibiotic Susceptibility of TB Is Reflected in Sputum Beta Diversity

**Figure 3:**
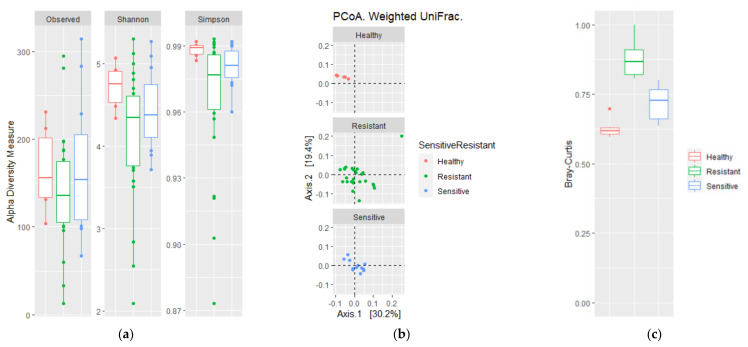
Comparison of alpha and beta diversity of drug-sensitive and drug-resistant TB patients. (**a**) Alpha diversity based on the number of observed species and Shannon and Simpson diversity; (**b**) PCoA weighted UniFrac; and (**c**) Bray–Curtis dissimilarity index. Plots represent HV samples (red symbols), drug-resistant TB patients (green), and drug-sensitive TB patients (blue). Statistical significance between groups is indicated by (*p* < 0.05). Wilcoxon and ADONIS tests for alpha and beta diversity comparisons, respectively, were done.

To evaluate if the differences in antibiotic susceptibility of *M. tuberculosis* were reflected in the microbial diversity of the sputum microbiota, α- and β-diversity of these three groups were analyzed in a multigroup comparison (Figure 3 and Figure S2). No differences were found for previously mentioned α-diversity indexes, although the Simpson index (*p* = 0.063) was lower and had a higher dispersion in DR-TB patients, whereas DS-TB and HVs tended to be similar (Figure 3a). However, measurements of β-diversity, both weighted and unweighted UniFrac distances (*p* = 0.013 and *p* = 0.006, respectively) and the BC index (*p* = 0.001) revealed significant variation between DR-TB and DS-TB patient bacterial communities compared to HVs (Figure 3b,c). Thus, although no differences in alpha diversity were observed between the respiratory tract microbiota of DR-TB and DS-TB patient subgroups, differences in richness, abundance, and phylogenetic diversity were observed when the three groups were compared (Figure 3b,c).

### 3.4. Microbiota Composition of TB Patients and HVs

**Figure 4:**
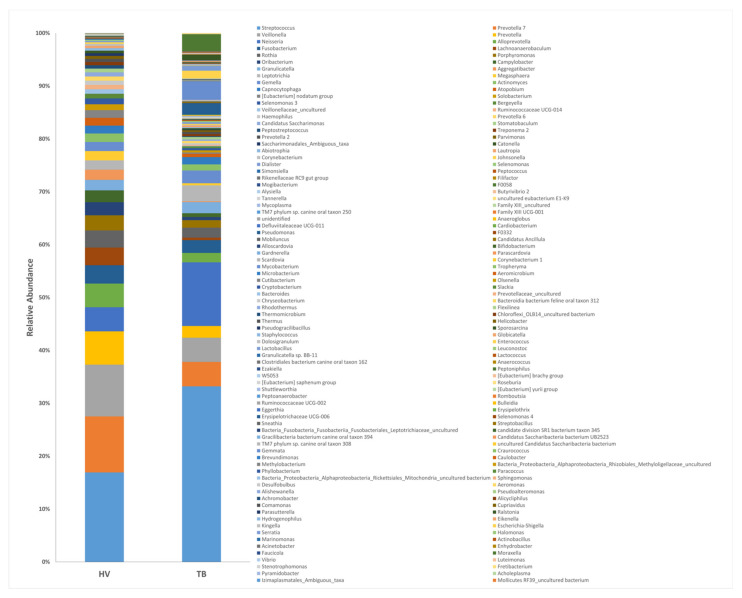
Composition of the dominant genera in the sputum of TB patients and healthy volunteers. 16S rRNA hypervariable regions V3–V4 from sputum samples were sequenced, and the relative abundance of genera is shown.

To understand the phylogenetic diversity in the lungs of TB patients, the composition of the respiratory tract microbiota in these groups was analyzed and then searched for differentially abundant taxa using the Analysis of Compositions of Microbiomes with Bias Correction (ANCOM-BC) methodology [27]. TB patient and HV respiratory tract microbiomes were composed of 202 independent identifiable genera, of which 142 and 70 were detected at a relative abundance threshold equal or higher than 0.01% and 0.1%, respectively, in TB patients; in contrast, 72 and 53 genera were identified at 0.01% and 0.1% abundance in HVs. An increased presence of phylum Bacteroidetes Epsilonbactereoarchaeota, Patescibacteria, and Spirochaetes was observed in HVs compared to TB patients (log fold change (LFC) of 1.2, 1.8, 2.2, and 2.15, respectively) (Figure 4). The most abundant bacterial genus observed in the healthy volunteers’ group were *Streptococcus*, *Prevotella 7*, *Veillonella*, *Prevotella*, and *Alloprevotella* (15.44%, 9.66%, 8.94%, 5.74%, and 4.04% respectively), whereas *Streptococcus*, *Neisseria*, *Prevotella 7*, *Moraxella*, and *Veillonella* (31.18%, 11.31%, 4.34%, 4.17%, and 4.86%) were the predominant genus observed in the TB group (Figure 4).

**Figure 5:**
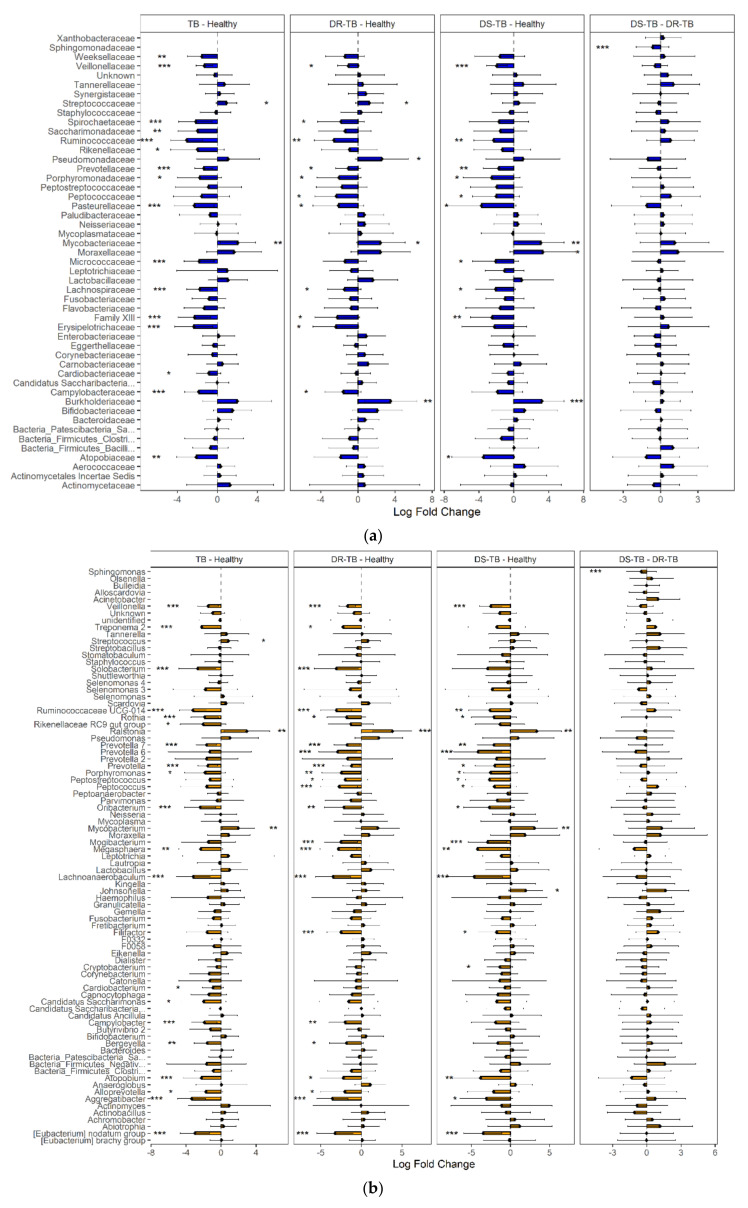
Differential abundance in sputum composition among HVs, DR-TB patients, and DS-TB patients. ANCOM-BC analysis showed differential abundance between TB vs. HV, DR-TB vs. HV, DS-TB vs. HV, and DR-TB vs. DS-TB at the family level (**a**) and the genus level (**b**). Statistical significance between groups is indicated by *, ** and ***, corresponding to *p* < 0.05, <0.01 and <0.001 respectively.

Using ANCOM-BC analysis, 18 bacterial families with differential abundance between TB patients and HVs and 23 genera were identified (Figure 5). HVs were characterized by an increased abundance (LFC > 2) of anaerobic genera: *Aggregatibacter* (previously known *as Actinobacilus*), *Ruminococcaceae UCG014*, *Lachnoanaerobaculum*, Eubacterium no datum group, *Solobacterium*, *Oribacterium*, *Megasphaera*, *Atopobium*, *Rothia*, *Porphyromonas*, and *Treponema 2*, which are also found in oral commensal microbiota. Conversely, anaerobes contributed proportionally less to the relative abundance of the respiratory tract microbiota of TB patients (0.15%) compared to that of HVs (0.398%).

We hypothesized that *M. tuberculosis* resistance to antibiotic treatment could modify or reflect respiratory bacterial ecology, thus resulting in different microbial configurations. Comparative analysis of these groups (Figure 5a) showed that more than 90% of the taxa identified in both DR-TB and DS-TB correspond to Firmicutes (46.97% and 51.85%), Proteobacteria (23.73% and 18.61%), Bacteroidetes (11.88% and 13.31%), and Actinobacteria (8.40% and 6.76%). The most abundant bacterial genera (Figure 5b) observed in both TB subgroups were *Streptococcus*, *Neisseria*, *Veillonella*, and *Prevotella 7* (29.75% and 34.3%, 11.16% and 11.63%, 4.84% and 3.08%, and 4.23% and 4.57%, respectively). When DR-TB or DS-TB microbiota were compared to HVs, 23 and 21 differentially abundant genera were found. Higher diversity and heterogeneity were found in DR-TB individuals (Figure 3b and Figure 5b). However, unlike a recent study [31] that found differences in diversity and an increased abundance of the genera *Leptotrichia*, *Granulicatella*, and *Campylobacter* in mono-resistant TB vs. DS-TB, we only found members of the *Sphingomonadaceae* family significantly more abundant in DR-TB patients than DS-TB patients.

### 3.5. Core Lung Microbiota Composition

**Figure 6:**
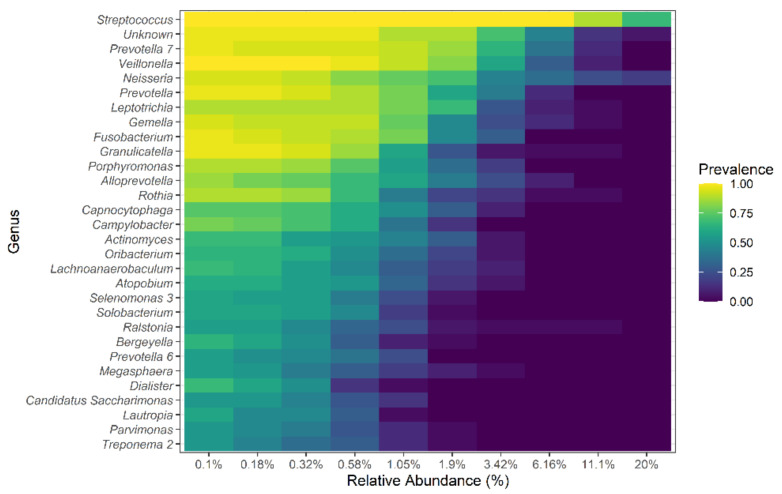
Core lung microbiome at the genus level. The prevalence and relative abundance of the core microbiome at the genus level of both TB patients and HVs. The top 30 genera are shown.

To explore the overall composition of the respiratory tract microbiome and its changes during TB, a core microbiota was identified (Figure 6). This core microbiome consisted of all bacterial genera present in at least 80% of the samples of each group (TB patients and HVs) and at a relative abundance of more than 0.1%. Analysis of the microbiome of both TB patients and HVs reveal a core respiratory tract microbiome that consisted of Firmicutes, Bacteroidetes, Proteobacteria, Fusobacteria, and Actinobacteria. These phyla represented over 95% of the identified taxa. Eleven genera composed this core respiratory tract microbiome, including *Streptococcus*, *Neisseria*, *Gemella*, *Granulicatella*, *Prevotella*, *Prevotella-7*, *Veillonella*, *Fusobacterium*, *Rothia*, *Porphyromonas*, and *Alloprevotella*. Ten additional genera were identified as part of a HV, but not TB, core microbiome. *Ralstonia* spp. (an emerging respiratory tract pathogen), *Moraxella* spp., as well as *M tuberculosis* were exclusively found in TB patients’ microbiomes, and *Alysiella* spp. and *Eubacterium* E1-K9 were only found in HVs and not in TB patients. Six members of the core, namely, *Prevotella*, *Prevotella-7*, *Veillonella*, *Rothia*, *Porphyromonas*, and *Alloprevotella*, showed lower abundance in TB patients than in HVs, and only *Streptococcus* was present at a higher abundance in TB patients.

## 4. Discussion

The microbiota of the respiratory tract is increasingly being recognized as an important component of respiratory health, and it has been associated with susceptibility to infection [15,16,32]. Understanding the composition and function of the microbiome is fundamental for the evolution of new therapeutic approaches and a better understanding of the development and establishment of multifactorial diseases such as tuberculosis.

Unlike the widely studied gut microbiota, research on lung-associated microbiota has only just started. Among the reasons for this are technical constraints to obtaining compatible samples from both patients and healthy volunteers, the low bacterial biomass, and the previous idea that the lower respiratory tract is sterile. However, recent studies have shown significant differences between healthy individuals and COPD, asthma, idiopathic pulmonary disease, cystic fibrosis, and TB patients [17,33,34].

Several studies of the microbiome of TB patients compared to healthy individuals have been published; however, the results are not consistent, which reveal differences in populations and also distinct experimental protocols that include differences in the type of sample (bronchoalveolar lavage vs. sputum), 16S rRNA gene hypervariable region sequenced, and differences in the chosen experimental controls [4,18,19,20,21,22].

The present study aimed to identify differences in the composition and diversity of microbes among TB patients and healthy individuals and examine the respiratory tract bacterial ecology of TB patients with differing *M. tuberculosis* bacillus resistance to antibiotics. Sputum samples, which represent a mixture of upper and lower respiratory tract microbiota, were chosen because of the non-invasive methodology that can be ethically collected from healthy volunteers with minimal modifications.

The microbial composition of the sputum in pulmonary tuberculosis patients was more diverse and dissimilar than that of healthy volunteers, which was highly homogenous, consistent with earlier investigations in Chinese and Indian populations [18,19]. These and other studies have indicated that the healthy respiratory tract harbors a mixture of microbes from the upper respiratory tract and the oral cavity [35]. In agreement, the most abundant bacterial genus in HVs and TB patients also corresponded to known oral microbiota: Gram-positive *Streptococcus* and Gram-negative *Moraxella*, *Neisseria*, *Veillonella*, and *Prevotella*. Thus, we speculate that in the healthy lung, the host is constantly discerning between commensals and pathogens through the adaptive immune response; however, during *Mtb* infection, the increased local inflammatory response and tissue damage results in a lung environment that is more susceptible to the colonization of foreign microorganisms [36].

In order to identify differentially abundant taxa in TB patients compared to HVs, we used ANCOM-BC, a methodology of differential abundance analysis for microbial absolute abundance data [27], which estimates the unknown sampling fractions and corrects the bias introduced by their differences among samples. *Streptococcus*, *Prevotella 7*, and *Veillonella* were among the five dominant genera in both groups. However, TB patients also had *Neisseria* and *Moraxella*, whereas *Prevotella* and *Alloprevotella* were among the most prevalent in the HV group. The high prevalence of these genera in the respiratory tract microbiota is consistent with other reports [18,19,20].

The genera *Moraxella* and *Ralstonia* were found exclusively in TB patients. *Moraxella* spp. are obligate aerobic Gram-negative bacteria, normal constituents of the oral microbiome and other mucosal membranes but opportunistic pathogens of the lower respiratory tract [37]. *M*. *catarrhalis* has been associated with lung infections, particularly in elderly or immunosuppressed individuals [38]. The genus *Ralstonia* are aerobic Gram-negative non-fermenting bacteria that are also part of the normal oral and upper respiratory tract microbiome and an emerging opportunistic pathogen of the lower respiratory tract. The most frequent species include *R*. *pickettii* and *R*. *insidiosa*, identified in nosocomial infections [39,40,41]. These microorganisms probably reach the lungs from the oral cavity through micro-aspiration and a damaged mucosal barrier.

Overall, TB patients’ sputum was characterized by a decreased abundance of Bacteroidetes and an increase in Proteobacteria. Furthermore, seven genera of the core microbiome were identified, having a significant change in relative abundance between HVs and TB patients, namely, *Prevotella*, *Prevotella_7*, *Porphyromonas*, *Alloprevotella*, *Veillonella*, *Rothia*, and *Streptococcus*; of these, all but *Streptococcus* are more abundant in healthy individuals than in TB patients. *Prevotella* and *Veillonella* spp. have been demonstrated to produce short-chain fatty acids (SCFAs), enhance immunity, and suppress the inflammatory response in other mucosal surfaces [42,43,44]; in contrast, *Streptococcus* is a common opportunistic pathogen of the lower respiratory tract. These data suggest that *M. tuberculosis* infection contributes to a change in the lung microbiota. Another possibility is that a microbiome with a reduced diversity favors the establishment of *M. tuberculosis* infection.

One of the major motivations for identifying a ‘common core’ across TB patients and HVs is to define components of the microbiome that may be particularly significant in the host–microbiome interaction. The common core members of the microbiota included Bacteroidetes, Firmicutes, Proteobacteria, and, in lower relative abundance, Actinobacteria, and Fusobacteria. Eleven genera comprised this nuclear microbiome, including *Streptococcus*, *Veillonella*, and *Granulicatella*, which were also identified as members of the core lung microbiome in Indian and Chinese studies [18,19,22]; thus, they may represent nuclear members of the respiratory tract microbiome and not only a geographical, ethnic, or socioeconomic characteristic of our group of study.

Finally, changes in the bacterial ecology between drug-sensitive (DS-TB) and drug-resistant (DR-TB) *Mtb* infection revealed significant differences in the abundance of the *Sphingomonadaceae* family. The *Sphingomonas* spp. is naturally resistant to aminoglycoside antibiotics; thus, antibiotic treatment may inadvertently select for these species. Moreover, *Sphingomonas paucimobilis* has been recently identified as an emerging opportunistic respiratory tract nosocomial pathogen [43,44]. However, when the three groups, DS-TB, DR-TB, and HVs, were compared, 23 genera were found to have differential abundance, but five genera (*Alloprevotella*, *Bergeyella*, *Campylobacter*, *Solobacterium*, and *Treponema* 2) were significantly different only in DR-TB and two (*Johnsonella* and *Cryptobacterium*) only in DS-TB. Most of these genera correspond to upper respiratory tract commensal microorganisms that may gain access to the lung due to mucosal barrier damage caused by *Mtb* infection.

We think that the above conclusions are sound and useful despite the inherent limitations of our study. One of them is the use of sputum samples; whereas bronchoalveolar aspiration samples can provide the closest resemblance to the pulmonary ecosystem, ethical considerations preclude us from using them in most cases, especially in HVs. Sputum samples have been regarded as representative of upper airways and useful for insight into the respiratory microbiome [4,16,18,19]. Moreover, sputum is used clinically as an effective surrogate for airway samples, despite acknowledged contamination from the upper airways and saliva [45]. The small number of HVs, with significant differences in age and gender, is another limitation of our work, but these variables were considered covariates for multiple testing comparisons in our ANCOM-BC model.

Future studies should include longitudinal analysis, where lung microbiota and the immune response of TB patients are analyzed throughout treatment to provide insight into lung microbiome dynamics during infection and the effect of different treatments.

## 5. Conclusions

In conclusion, our data show that a healthy respiratory tract is composed of a relatively homogenous bacterial community, while TB patients had a significantly more diverse and dissimilar bacterial assembly. In this study, the TB sputum microbiome harbored 7 phyla and more than 200 genera, including obligate and facultative anaerobes, oral bacteria, and opportunistic pathogens. Our data suggest that *Mycobacterial* infection potentially modifies the lung microenvironment or the mucosal barrier, allowing the colonization and expansion of more diverse taxa, including commensal bacteria from the upper respiratory tract and the oral cavity. Changes in the composition of the microbiota and, therefore, the local immune response could determine the treatment and, perhaps, the severity of lung parenchyma damage.

## Figures and Tables

**Table 1:** Demographic and clinical characteristics of the study participants.

Clinical Variables Subjects (*n*)	TBP 39	HVs 6
Age median (years)	37 (20–82)	28 (23–55)
Gender (Male: %)	65%	20%
Pulmonary TB Classification (%)	48.7% (*n* = 19) BK3 7.7% (*n* = 3) BK2 10% (*n* = 4) BK1 15% (*n* = 6) BK0	-
Place of Residence		100%
Mexico City Metropolitan Area Countryside States (Guerrero, Veracruz, Puebla, Quintana Ro) (%) *M. tuberculosis* positive culture (%)	3% (18,7,4,4,3%) 100%	-
Antibiotic-resistant *M. tuberculosis* (%)	69.2% (*n* = 27)	-
Previous TB Treatment (%)	45.1%	-
HIV seropositive (%)	0	0

Abbreviations: TBP = tuberculosis patients, HVs = healthy volunteers, BK = bacilloscopy, HIV = human immunodeficiency virus.

## Data Availability

The data that support the findings of this study are available from the corresponding author, E.S.-H., upon reasonable request.

## Supplementary Materials

The following are available online at https://www.mdpi.com/article/10.3390/microorganisms9081762/s1, Figure S1: Bacterial diversity in sputum samples; Figure S2: Composition of sputum microbiota of TB patients and healthy volunteers.
