# Supplementary material for: Altered Microbial Composition of Drug-Sensitive and Drug-Resistant TB Patients Compared with Healthy Volunteers
Source: Microorganisms. 2021 Aug 18;9(8):1762. doi: 10.3390/microorganisms9081762 (PMC8398572; doi:10.3390/microorganisms9081762)
Supplement: Supplementary file 1 [file microorganisms-09-01762-s001.zip › microorganisms-1323368-supplementary.pdf]

microorganisms

MDPI

Supplementary Material

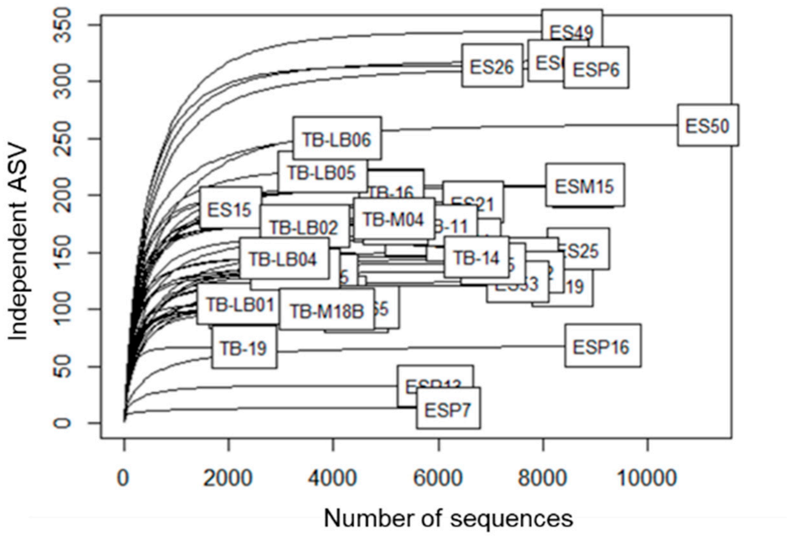

Figure S1. Bacterial diversity in sputum samples. The graph shows rarefaction curves, indicating the number of independent ASVs in relation to the number of 16S rRNA sequences. TB patients are labeled TB# and ES#; healthy volunteers are labeled TB-LB#.

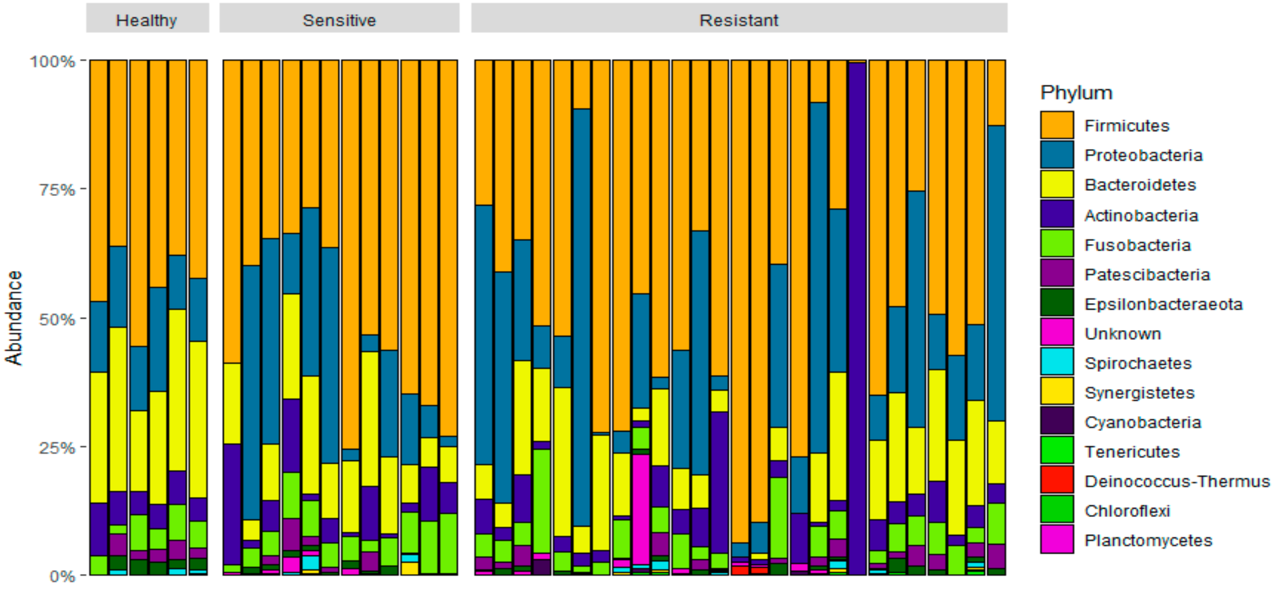

(**a**)

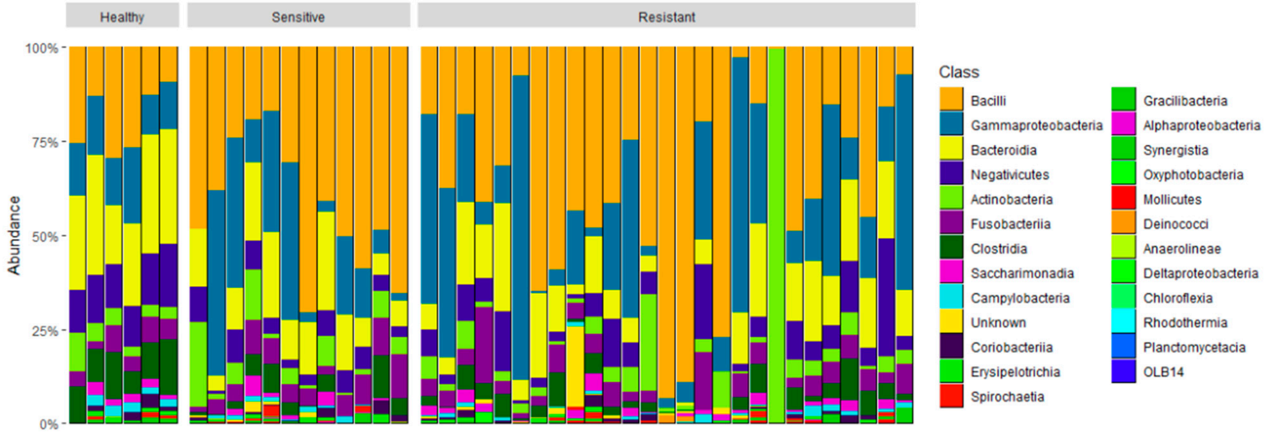

(**b**)

Figure S2. Composition of sputum microbiota of TB patients and healthy volunteers. 16S rRNA hypervariable regions V3–V4 from sputum samples were sequenced, and relative abundances for the individual samples are shown. (**a**) Phylum and (**b**) class of healthy volunteers (Healthy), drug-sensitive TB (Sensitive), and drug-resistant TB (Resistant) are shown.
